# Magnetic seed *versus* guidewire-based breast cancer localization with magnetic lymph node detection: cost-minimization analysis

**DOI:** 10.1093/bjs/znaf253

**Published:** 2025-12-01

**Authors:** Eirini Pantiora, Filipa Sampaio, Allan Jazrawi, Fredrik Wärnberg, Staffan Eriksson, Andreas Karakatsanis

**Affiliations:** Department of Surgical Sciences, Uppsala University, Uppsala, Sweden; Section for Breast Surgery, Department of Surgery, Uppsala University Hospital, Uppsala, Sweden; Department of Public Health and Caring Sciences, Uppsala University, Uppsala, Sweden; Centre for Clinical Research, Department of Surgical Sciences, Uppsala University, Västerås, Sweden; Section for Breast Surgery, Department of Surgery, Västmanlands County Hospital, Västerås, Sweden; Sahlgrenska Centre for Cancer Research, Department of Surgery, Institute of Clinical Sciences, Sahlgrenska Academy, University of Gothenburg, Gothenburg, Sweden; Department of Surgery, Sahlgrenska University Hospital, Gothenburg, Sweden; Centre for Clinical Research, Department of Surgical Sciences, Uppsala University, Västerås, Sweden; Section for Breast Surgery, Department of Surgery, Västmanlands County Hospital, Västerås, Sweden; Department of Surgical Sciences, Uppsala University, Uppsala, Sweden; Section for Breast Surgery, Department of Surgery, Uppsala University Hospital, Uppsala, Sweden

## Abstract

**Background:**

Magnetic seeds have comparable performance to guidewires in breast lesion localization with the advantages of shorter operating time, facilitated logistics, and higher staff satisfaction. However, the high cost of the device remains a concern and warrants health economic evaluation.

**Methods:**

This is a predefined health economic analysis of a pragmatic RCT including 426 patients (median age of 65 (interquartile range (i.q.r.) 56–71) years, median BMI of 26.6 (i.q.r. 24.0–29.8) kg/m^2^, and a median tumour size of 11 (i.q.r. 8–15) mm) with non-palpable breast cancer, randomized to localization of the tumour with either a magnetic seed or a guidewire. Sentinel lymph node detection was performed using superparamagnetic iron oxide nanoparticles, enabling a totally magnetic approach. A cost-minimization analysis was conducted, from a healthcare system perspective, using unadjusted and adjusted analyses of costs.

**Results:**

The unadjusted analysis did not show any difference in incremental costs (guidewire €3337 *versus* magnetic seed €3274; difference −€63 (95% c.i. −€302 to €174); *P* = 0.599). The adjusted analysis, including marker, type of breast surgery performed, and single-session lesion and SLN localization, showed that the magnetic seed was associated with reduced costs (guidewire €3514 *versus* magnetic seed €3123; difference −€391 (95% c.i. −€422 to −€360); *P* = 0.002), corresponding to a 11.1% reduction. Sensitivity analyses did not change direction of outcome.

**Conclusion:**

In this predefined health economic analysis of an RCT, the use of magnetic seeds resulted in incremental cost containment, despite the increased cost of the device. Contributing factors included shorter localization time, shorter operating time, and process streamlining.

## Teaser

This predefined economic analysis of the MAGTOTAL RCT compared magnetic seed with guidewire localization for non-palpable breast cancer in the context of magnetic sentinel node biopsy. Despite the higher device cost, magnetic seed localization was associated with lower overall procedural costs due to shorter operating times and streamlined logistics. These findings support the cost-efficiency and operational advantages of a totally magnetic surgical approach.

## Introduction

Breast-conserving surgery (BCS) with preoperative lesion localization and sentinel lymph node biopsy (SLNB) has become the mainstay treatment of early-stage, non-palpable breast cancer^1,2^. Guidewire localization has been the standard since the introduction of BCS^3^. While affordable and accessible, it poses scheduling challenges, as the wire has to be inserted on the day of surgery. The need to decouple preoperative localization from surgery led to wireless localization devices such as probe-detectable seeds or tags that can be placed days before surgery, all yielding favourable outcomes, but with a higher device cost^4–9^. At the same time, SLNB has traditionally relied on the use of a radioisotope, which has a high identification rate, but is restricted by a short half-life, stringent regulations, the need of nuclear oversight, and recurring supply shortages^10,11^.

A 5-mm ferromagnetic marker for lesion localization (Magseed^©^, Endomag, UK) and a superparamagnetic iron oxide (SPIO) nanoparticle suspension for SLNB (Magtrace^©^, Endomag, UK) have been extensively studied and adopted in clinical routine^12–15^. Recently, the MAGTOTAL RCT compared a totally magnetic technique (magnetic seed for the tumour, SPIO for SLN) with guidewire and SPIO. In this RCT the main outcomes of volumes excised, re-excision rates, and complications were equivalent, but the magnetic seed resulted in less localization failures, shorter operating time, and higher preference by healthcare practitioners^16^.

As cost-effectiveness is crucial for the evaluation and selection of new surgical technologies, the aim of this predefined secondary health economic analysis of the MAGTOTAL RCT was to provide insights on the economic impact of the magnetic marker against the previous standard of the guidewire.

## Methods

### Study design

This study is a within-trial health economic evaluation, conducted from a healthcare system perspective, based on data from the MAGTOTAL RCT^16^. This pragmatic RCT was conducted in three hospitals in Sweden between 1 May 2018 and 1 May 2022. Seed placement and SPIO injection could be done by either a breast radiologist or a surgeon during the preoperative consultation, whereas guidewire placement could only be done by a radiologist. The trial was approved by the Uppsala Regional Ethics Committee and registered at ISRCTN (ID: ISRCTN11914537). The present work is reported according to the Consolidated Health Economic Evaluation Reporting Standards (CHEERS) statement^17^.

### Study population and procedure

The trial included adults with non-palpable cT_is_–T_3_ N0 breast cancer, scheduled to undergo BCS and SLNB, randomly allocated (1 : 1 ratio) to either a magnetic seed or guidewire for tumour localization with all patients undergoing SPIO-guided SLNB. A full report on inclusion criteria is reported in the published results of the trial^16^. All patients provided written informed consent.

### Comparators

The magnetic seed is compared with the guidewire that is the standard-of-care localization method. The wire used in the trial was the Hawkins™ Hardwire BLN with echogenic tip (Argon^©^, USA). The costs per device were €278 and €38 respectively.

### Data collection

#### Sample characteristics

Patient age, BMI, and tumour characteristics (laterality, size, histology, subtype), type of radiological workup, and receipt of primary systemic therapy were collected as baseline characteristics. Preoperative volumetry was performed to define the optimal resection volume (ORV), defined as the volume required to remove the tumour with 1-cm margins. Localization time and personnel, time from localization to surgery, operating time, and type of surgery were prospectively documented.

The primary outcomes were positive margins and the resection ratio (volume excised/ORV) in patients with negative margins. Secondary outcomes included successful SLNB, adverse events, failed localizations, operating time, and ease of implementation by healthcare practitioners. Patient-reported outcome measures (PROMs) and patient-reported experience measures (PREMs) were also collected and will be reported elsewhere.

#### Resource use and costs

Costs were estimated from a Swedish healthcare system perspective, which is universal and publicly funded, with each region as the payer. The cost analysis used a bottom-up approach (micro-costing) to accurately identify, measure, and assign value to specific cost items, such as the cost of the device, cost of personnel time, and operating theatre costs^18^.

The trial results showed higher satisfaction of surgeons, radiologists, and operating theatre coordinators with the magnetic seed^16^. For the coordinators, the main reason was that, when a patient would receive a guidewire, it would lead to rescheduling of the theatre list to avoid (i) either one late start (meaning an average theatre delay by 90 min (accounting for lesion localization, transfer from radiology to the day-surgery department, admission and preparation for anaesthesia)) for every fifth operating list, or (ii) that the patient receiving a guidewire would have to wait more days from the preoperative appointment to surgery. The first would affect productivity, whereas the latter would risk breaching the interval between diagnosis and treatment required by the Standardized Treatment Pathway (Standardiserad Vårdförlopp (SVF)) implemented in Sweden for all cancer patients^19^. The extra time required for rescheduling was estimated to correspond to a total of 1 h for each of the three coordinators.

**Fig. 1:**
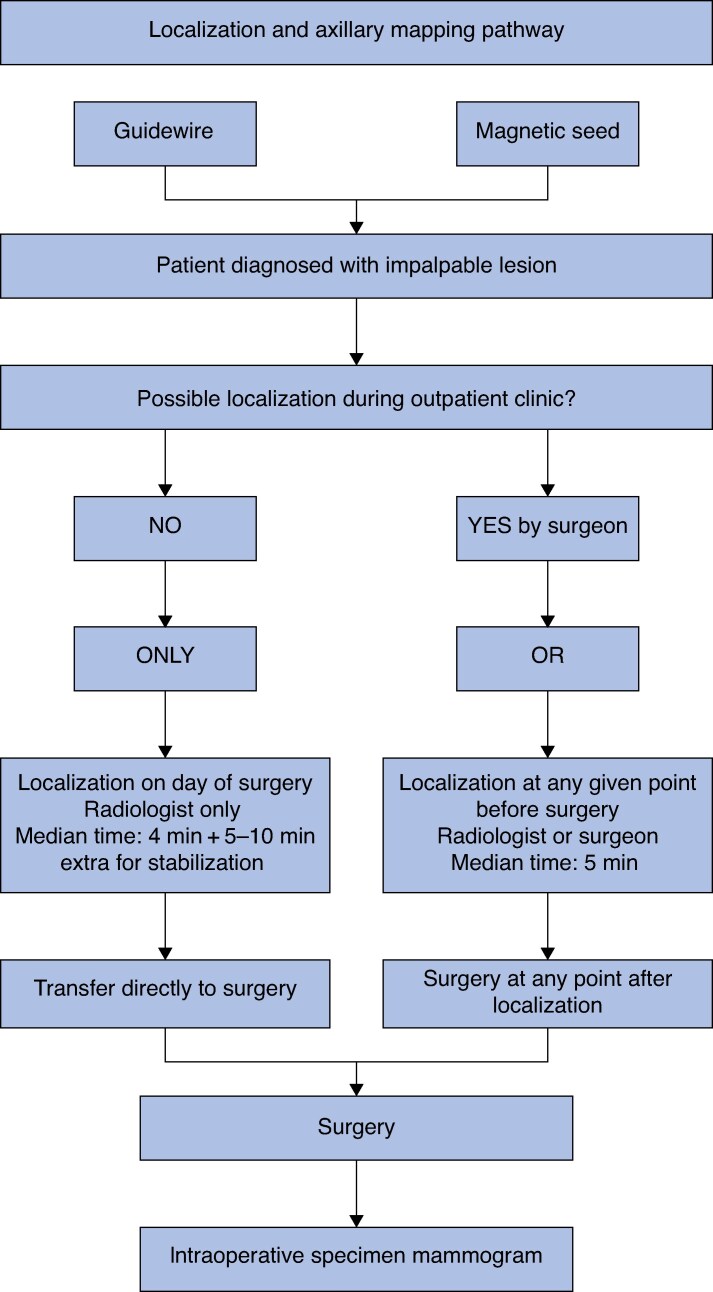
Flow chart summarizing the pathway for the magnetic marker and the guidewire

The trial protocol allowed for the implementation of local routines in the localization procedure, leading to variability in the timing and setting of magnetic seed placement and SPIO injection. Seed localization could be performed by the surgeon under ultrasonographic guidance during preoperative planning, whereas SPIO injection could be performed by the surgeon under ultrasonographic guidance or by freehand. When localization was performed by a radiologist, this was done under ultrasonographic or stereotactic guidance, with the aid of a radiology nurse. Guidewire placement required an additional 5–10 min to stabilize and secure the guidewire, a procedure not required in magnetic seed localization. In both arms, post-localization mammograms were performed to ensure correct placement. Intraoperatively, the guidewire was identified through direct visual inspection, whereas the magnetic seed was localized with the same probe that was used for SLNB (Sentimag^©^, Endomag, UK). Intraoperative specimen radiology was performed in all cases to ensure the presence of the marker and the lesion in the excision specimen, and cavity shavers were taken when needed. The workflow is illustrated in *Fig. 1*.

The cost of the additional time of healthcare professionals was estimated, considering the hourly salary of all healthcare personnel involved sourced from salary logs including employee insurance benefits. SPIO for SNLB was already routinely used in all three hospitals and included for both trial arms, thus its cost was not included in this analysis^20^. Finally, despite more failed localizations in the guidewire arm, these instances were not included in the analysis, as wires were either replaced with magnetic seeds or, in cases of intraoperative dislocation, the peritumoral magnetic signal and the tissue discoloration from SPIO guided the excision.

Information on the cost of each device was obtained by reviewing invoices from the recruitment interval to capture the pricing. Information regarding deployment and surgical operating times required for each procedure was prospectively documented. The time spent preparing for localization, as well as the time required to perform post-localization mammogram, clean up the room, and register the procedure, was equal between the two methods and therefore not included in the analysis. Localization and operating time data were included in the RCT outcomes. Respective hourly salaries were used to estimate the cost of the time of the two techniques. The cost/min for operating theatre use is routinely registered in the hospital operational system and was retrieved from the patient logs.

Total costs were estimated including material costs (cost of device), the deployment costs, and operating theatre time. Costs were collected in 2022 Swedish krona and converted into 2022 € using the Evidence for Policy & Practice Information (EPPI) cost conversion database^21^. All resources used, unit costs, and total costs are shown in *Table 1*.

**Table 1:** Resources, unit costs, and total costs

	Magseed			Guidewire			Source of unit cost
	Frequency	Unit cost	Total (€)	Frequency	Unit cost	Total (€)	
**Material costs**							
Device cost	215	278	59 770	208	38	7904	Per invoice
Delivery costs	NA	NA	NA	NA	NA	NA	Per invoice
**Deployment cost**							
Radiology (physician + nurse)	203 × 4 min	1.7/min	1362.1	208 × 15 min	1.7/min	5304	Hourly rate of consultant breast radiologist from salary logs
Surgeon time	12	0	0	NA	NA	0	Performed during preoperative consultation
Referral to radiology	203	265	53 795	208	265	55 120	Hospital invoicing system
Capital	0	0	0	0	0	0	
**Operating theatre list planning**							
Surgical coordinators’ time	0	0	0	42*	96.11	4036.6	Hourly rate of three breast coordinators from salary logs
Operating time (median)	215 × 69 min	37.1/min	550 378.5†	208 × 75.5 min	37.1/min	582 618.4†	
Total cost (excluding device)			605 535.6			647 079	
Total cost (including device)			665 305.6			654 983	

All costs are in €, 2022. Time is provided in minutes; respective monetary costs are multiplied by the respective cost/min. *For the surgical coordinator time, the extra time required for the guidewire responds to every fifth patient and corresponds to one working hour for three breast nurses/operating theatre coordinators. †Number of procedures multiplied by procedural time multiplied by cost/min. NA, not applicable.

Given that the nature of the intervention did not have long-term effects, the time horizon of the analysis was the interval from lesion localization and axillary mapping, and included the immediate postoperative interval, which is universally defined as 30 days^22^.

### Data analysis

The trial reported equivalence for the primary outcomes of re-excision (per protocol analysis 2.91% for guidewire *versus* 2.84% for magnetic seed; difference 0.07% (95% c.i., −3.10% to 3.30%); *P* = 0.950) and resection ratio (median of 1.93 (interquartile range (i.q.r.) 1.18–3.43) *versus* median of 2.01 (i.q.r. 1.11–3.47); *P* = 0.700). No differences were demonstrated in complication rates (7.3% for guidewire *versus* 9.8% for magnetic seed; difference −2.5% (95% c.i., −3.3% to 8.3%); *P* = 0.449). Despite the fact that failed localizations were more frequent with the guidewire than the magnetic seed (10.1% *versus* 1.9%; difference 8.2% (95% c.i. 3.3% to 13.2%); *P* < 0.001), the peritumoral SPIO injection could guide specimen resection^16^. Moreover, preliminary data analysis on quality of life and PROMs have not shown any differences between the two methods^23^. These results satisfy the assumption of clinical equivalence that underpinned the use of a cost-minimization framework for the present economic analysis.

#### Descriptives

Continuous variables are summarized as mean(s.d.), mean (95% c.i.), or median (i.q.r.), as appropriate. Bootstrapping with 1000 iterations was performed to account for uncertainty and the subsequent means and medians are presented with 95% confidence intervals. Unadjusted comparisons were performed using linear regression and the marginal differences with 95% confidence intervals are reported.

#### Analysis of cost data

To identify the cost of the implementation of the magnetic seed, regardless of the perceived ease of logistics, a stepped procedure was adopted comparing the cost analyses with the monetary costs associated with operating theatre scheduling. The additional covariates included in the adjusted analysis (apart from type of marker), that is type of breast surgery (simple wide local excision (WLE), level I oncoplastic breast-conserving surgery (OPBCS), or therapeutic mammaplasty/mastopexy (TM)) and whether lesion localization and SLN mapping occurred in a single session, were pre-specified as key procedural factors shown to influence operating theatre time and overall cost, based on the primary trial analysis^16^. A stepwise generalized linear model was employed to fit the cost data using a gamma distribution and a log-link. *P* < 0.050 (two-sided) was considered statistically significant. SPSS^®^ (IBM, Armonk, NY, USA; version 28) and Stata (version 17) software were used for the analysis.

### Sensitivity analysis

The following deterministic sensitivity analyses were performed: incremental costs if all localizations had been performed by radiologists; and incremental costs if all the magnetic seeds in patients that were deployed under ultrasonographic (but not stereotactic) guidance had been performed by surgeons. These two scenarios reflect the predominant routine practice patterns in the USA, the UK, and large parts of Europe and in Central Europe, such as Germany, Austria, and Switzerland, respectively^24^.

## Results

Detailed trial results have been reported elsewhere^16^. The population comprised 426 patients (median age of 65 (i.q.r. 56–71) years, median BMI of 26.6 (i.q.r. 24.0–29.8) kg/m^2^, and median tumour size of 11 (i.q.r. 8–15) mm) and the main characteristics are summarized in *Table 2*.

**Table 2:** Trial population characteristics

	Allocation arm		*P*
	Guidewire	Magnetic marker	
Age (years), median (i.q.r.)	67 (56–72)	64 (56–69)	0.082*
BMI (kg/m^2^), median (i.q.r.)	26.1 (23.7–29.7)	26.7 (24.1–29.9)	0.332*
**Screening detected lesion**			0.859†
No	16 (7.8)	18 (8.9)	
Yes	188 (92.2)	194 (91.1)	
**Lateralization**			0.843†
Right breast	95 (48.7)	100 (47.4)	
Left breast	100 (51.3)	111 (52.6)	
Lesion size (mm), median (i.q.r.)	10 (8–15)	11 (8–15)	0.138†
**Type of surgery**			0.460†
WLE	180 (84.9)	169 (81.3)	
OPBCS	24 (11.3)	26 (12.5)	
TM	8 (3.8)	13 (6.3)	

Values are *n* (%) unless otherwise indicated. Results are presented per protocol. *Mann–Whitney *U* test. †Fisher’s exact test. i.q.r., interquartile range; WLE, wide local excision; OPBCS, oncoplastic breast-conserving surgery (corresponding to oncoplastic lumpectomy); TM, therapeutic mammaplasty/mastopexy.

Magnetic seeds were placed a median of 5 (i.q.r. 1–8) days ahead of surgery, with a median of 4 (i.q.r. 3–5) min required for the localization session, most often (189 of 215; 92.2%) under ultrasonographic guidance and as a single localization session (180 of 215; 85.3%) (*Table 3*).

**Table 3:** Patterns of lesion localization and SPIO administration

	Guidewire	Magnetic marker	*P*
**Localization modality**			0.708*
Ultrasonographic	194 (93.3)	189 (92.2)	
Stereotactic	14 (6.7)	16 (7.8)	
Time from localization to surgery (days), median (i.q.r.)	0 (0–0)	5 (1–8)	<0.001†
Time for lesion localization (min), median (i.q.r.)	10 (10–11)	4 (3–5)	<0.001†
**SPIO administration**			<0.001*
Surgeon	85 (40.6)	29 (13.5)	
Radiologist	123 (59.4)	186 (86.5)	
**Lesion localized by**			<0.001*
Surgeon	0 (0.0)	12 (5.6)	
Radiologist	208 (100.0)	203 (94.4)	
Time from SPIO injection to surgery (days), median (i.q.r.)	7 (0–15)	6 (1–8)	0.041†
**Single localization procedure (breast and axilla)**			<0.001*
Yes	74 (33.7)	184 (85.6)	
No	138 (66.3)	31 (14.4)	

Values are *n* (%) unless otherwise indicated. ‘Surgeon’ denotes freehand SPIO injection around the tumour. *Fisher’s exact test. †Mann–Whitney *U* test. SPIO, superparamagnetic iron oxide; i.q.r., interquartile range.

### Cost-minimization analysis

#### Base case analysis

The unadjusted analysis did not show any difference in incremental costs (guidewire €3337 *versus* magnetic seed €3274; difference −€63 (95% c.i. −€302 to €174); *P* = 0.599). The adjusted analysis, including marker (guidewire or magnetic seed), type of breast surgery performed (WLE *versus* OPBCS *versus* TM), and single localization (yes/no), showed that the magnetic seed was associated with reduced costs (guidewire €3514 *versus* magnetic seed €3123; difference −€391 (95% c.i. −€422 to −€360); *P* = 0.002), corresponding to a 11.1% reduction. The results are shown in *Table 4*.

**Table 4:** Cost-minimization analysis

	Unadjusted analysis			Adjusted analysis			
	Mean (95% c.i.)	Marginal difference (95% c.i.)	*P*	Coefficient (95% c.i.)	Marginal mean (95% c.i.)	Difference (95% c.i.)	*P*
**Localization device**							
Guidewire	3337 (3151,3524)	Reference category		Reference category	3514 (3333,3696)	Reference category	
Magnetic seed	3274 (3124,3160)	−63 (−302,174)	0.599*	−0.118 (−0.192,−0.044)	3123 (2973,3273)	−391 (−422,−360)	0.002†
**Type of breast surgery**							
WLE	3126 (3010,3241)	Reference category		Reference category	3137 (3024,3250)	Reference category	
OPBCS	3722 (3365,4078)	604 (144,1064)	<0.001*	0.156 (0.055,0.256)	3666 (3321,4010)	528 (297,760)	0.003†
TM	5232 (4560,5903)	2106 (1280,2932)	<0.001*	0.493 (0.342,0.643)	5135 (4387,5884)	1998 (1362,2634)	<0.001†
**Single localization session**							
Yes	3015 (2869,3161)	Reference category		Reference category	2988 (2820,3157)	Reference category	
No	3498 (3310,3686)	481 (243,720)	<0.001*	0.164 (0.087,0.240)	3519 (3361,3678)	531 (521,541)	<0.001†

Trial-based, unadjusted and adjusted cost-minimization analysis. Monetary units are €. The adjusted analysis was performed using a generalized linear model (using a gamma distribution and a log-link). *Regression analysis. †Generalized linear regression model. WLE, wide local excision; OPBCS, oncoplastic breast-conserving surgery, TM, therapeutic mammaplasty/mastopexy.

#### Sensitivity analyses

The sensitivity analyses did not change the results. Having a radiologist performing all the localizations would not have led to a cost difference (magnetic seed €3556 (95% c.i. 3406 to 3706) *versus* guidewire €3620 (95% c.i. 3433 to 3806); *P* = 0.601). If surgeons had placed all the magnetic seeds when ultrasonographic guidance was feasible, the cost difference would have been marginally in favour of the magnetic seed (€3287 (95% c.i. 3138 to 3439) *versus* guidewire €3618 (95% c.i. 3432 to 3805); *P* = 0.007). Full details are provided in *Table S1*. On the other hand, both adjusted sensitivity analyses (*Table S2*) still demonstrated that the magnetic seed was associated with incremental cost reduction.

## Discussion

The MAGTOTAL RCT corroborated previous observational data on the equivalent performance of magnetic seeds compared with guidewires with regard to clinical outcomes, with the advantages of decoupling lesion localization and SLN mapping from surgery and increased satisfaction from all stakeholders^7,12,25–27^. Furthermore, the magnetic seed was preferred by healthcare personnel, as it streamlined theatre planning procedures and increased productivity^16^.

Associated costs are always a concern during the implementation and adoption of modern technologies. In the base-case unadjusted analysis, no statistically significant cost difference was observed between arms, despite the significantly higher cost of the device per se. The incremental cost reduction associated with the magnetic seed suggests that the higher device cost was mitigated by the shorter operating theatre time, ease of planning, and the decoupling of lesion localization and SLN mapping from the day of surgery, in a single session and within a very wide time frame. The analysis did not include the cost of SPIO and the Sentimag^©^ probe, as they were used for SLNB in both trial arms. However, previous head-to-head comparisons have demonstrated reduced costs with SPIO compared with radioisotope, especially when administered before the day of surgery^14,15^. Moreover, the integration of SPIO for SLNB effectively eliminates the necessity for multiple devices, contributing to capital costs reduction and accelerated equipment depreciation. Interestingly, the only current alternative for single-probe lesion and SLN detection is the combination of radioisotope and radioactive seeds. This approach requires nuclear medicine oversight, thus posing access challenges, particularly in global settings, and incurs costs for transportation, storage, and disposal of radioactive materials^28,29^. Moreover, the short isotope half-life restricts preoperative localization and axillary mapping to the day of surgery or the afternoon before, offering less flexibility than the MAGTOTAL technique.

Further on, when the model was adjusted for predefined cost-driving covariates such as type of breast surgery and whether lesion localization and SLN mapping were performed in a single session, the magnetic seed arm demonstrated a significant 11.1% cost reduction. This change reflects the uneven distribution of these factors between groups, as more complex oncoplastic procedures and multisession workflows were associated with higher costs. Adjusting for these variables isolated the cost impact of the localization method itself, independent of surgical complexity or scheduling patterns. The direction and statistical significance of this finding were consistent across the deterministic sensitivity analyses, suggesting that the observed cost reduction is robust to different plausible workflow scenarios.

Numerous studies have demonstrated that wireless markers have comparable clinical outcomes and are logistically advantageous with a more sustainable environmental profile^4–6,30–36^. However, the existing literature remains deficient in addressing the financial implications of implementing wireless markers across different healthcare settings. This makes the study findings particularly relevant, as cost concerns remain the main barrier to adoption. While previous research has examined the cost-effectiveness of radioactive seeds, only one published study to date has assessed the budget impact of magnetic markers^28,29,37,38^. In Sweden, approximately two-thirds of new breast cancers are diagnosed via a screening programme and require preoperative localization^39^ . Acknowledging that this may not be the case internationally, the present analysis is specifically examining the incremental costs of a combined technique for lesion and SLN localization that is flexible and easily applicable, suggesting that appropriate reimbursement strategies could address practice variations, but still result in cost containment.

The main strength of the present study is that it is a predefined analysis stemming from a prospectively curated database of a pragmatic RCT, which minimizes selection or procedure bias. Moreover, the analysis captured the indirect costs that accompany breast lesion localization and the way these affect theatre planning and resource allocation. The use of magnetic seeds diminished the time theatre coordinators spent arranging the theatre lists to avoid delays related to same-day localization. Additionally, performing a cost-minimization analysis based on actual costs allowed for the avoidance of assumption-based models, which have been shown to affect the accuracy of health economy findings^40,41^.

Certain limitations need to be acknowledged. These findings are rooted in the Swedish healthcare context, which is publicly funded, regionally organized, and operates with high unit volumes. In this environment, the incremental device cost of the magnetic seed was offset by gains in operative efficiency and logistical flexibility. In other systems, cost implications may differ. In fee-for-service models, shorter operating times may not translate into direct cost savings for providers, but could improve throughput and revenue generation. In capitated or budget-constrained systems, efficiency gains may allow reallocation of theatre time to additional cases. In lower- and middle-income countries, barriers may include the availability of SPIO, access to magnetic probes, and the training of surgeons in ultrasound-guided placement. At the same time, whilst sensitivity analyses were performed, the potential effect of institutional experience and learning curves (particularly in centres transitioning from radiology-led to surgeon-led localization) on associated costs may not be fully captured. Nevertheless, the breakdown of the data may provide a basis for further studies in diverse settings. Finally, the potential impact of several factors on costs could not be directly captured. These include: the full effect of failed localizations, which were more frequent for the guidewire; the fact that patients who received a guidewire were not scheduled as first cases in the operating list to avoid operating theatre delays; and, finally, the fact that higher healthcare provider satisfaction with the magnetic seed was not monetized through a ‘willingness-to-pay’ approach.

This economic analysis showed that, despite higher device costs, magnetic seeds achieved overall cost containment within the frame of a totally magnetic technique through shorter localization and operating times, whilst increasing satisfaction among healthcare practitioners.

## Supplementary Material

znaf253_Supplementary_Data

## Data Availability

Enrolment in the trial started in May 2018, before the introduction of data sharing plans by the International Committee of Medical Journal Editors (ICMJE). The participants that consented to the trial did not give specific written consent for their data to be shared for purposes other than the trial and, according to the Swedish Ethical Review Authority, data sharing may be available after specific patient consent.
