# Supplementary material for: Magnetic seed versus guidewire-based breast cancer localization with magnetic lymph node detection: cost-minimization analysis
Source: Br J Surg. 2025 Dec 1;112(12):znaf253. doi: 10.1093/bjs/znaf253 (PMC12667277; doi:10.1093/bjs/znaf253)
Supplement: znaf253_Supplementary_Data [file znaf253_supplementary_data.zip › Protocol.docx]

Magnetic marker or guidewire localization of non-palpable breast cancer in combination with magnetic sentinel lymph node dissection. An open-label, phase 3, pragmatic, randomized controlled trial.

| Protocol ID Magnetic marker or Guide wire localisation of  non-palpable breast cancer in combination with  magnetic sentinel lymph node dissection. An open-  label, phase 3, pragmatic randomised controlled trial. |
| --- |
| Short title MAGtotal RCT |
| Date May 2018 |
| Principal investigator Andreas Karakatsanis, MD, PhD  Consultant Oncoplastic Surgeon, Associate Professor  Uppsala University Hospital, Uppsala, Sweden  andreas.karakatsanis@surgsci.uu.se |
| Co-investigators Fredrik Wärnberg, MD, PhD  Professor of Surgery  Department of Surgery,  University of Gothenburg, Sweden  fredrik.warnberg@vgregion.se |
| Staffan Eriksson, MD,PhD  Associate Professor of Surgery  Department of Surgery,  Västmanlands Hospital, Västerås, Sweden  staffan.eriksson@regionvastmanland.se |

**Contents**

Contents…………………………………………………………………………………………………………………………….3

List of Abbreviations…………………………………………………………………………………………………………..4

Summary ………………………………………………………………………………………………………………….……….5

1. Background
   1. Surgical Treatment of Non-palpable Breast Cancer…………….…………………….…………8
   2. Preoperative Lesion Localisation………………………………………………………………………….8
      1. Guidewire Localisation……………………………………………………………………………...8
      2. Non-Wire Localisation Methods………………………………………………………….…….8
      3. Ferromagnetic Seeds…………………………………………………………………………………9
   3. Sentinel Lymph Node Detection with the use of Superparamagnetic iron oxide nanoparticles………………………………………………………………………………………………….…..10
   4. The “MAGnetic marker To deTect primary Lesion and sentinel node in breast cancer” (MAGtotal) concept…………………………………………………………………………………………….10
2. Investigational Plan

2.1 Title……………………………………………………………………………………………………………………12

2.2 Objectives………………………………………………………………………………………………………….12

2.2.1 Primary Objective………………………………………………………………………..………….12

2.2.2 Secondary Objectives………………………………………………………………………………13

2.3 Patient Population and Indications………………………………………………………………….….13

2.3.1 Inclusion Criteria………………………………………………………………………………….….13

2.3.2 Exclusion Criteria…………………………………………………………………………………….13

2.4 Study Design………………………………………………………………………………………………………..14

2.5 Localisation Technique…………………………………………………………………………………………15

2.6 Sampling and Data Analysis Plan………………………………………………………………………….16

2.7 Statistical Analysis………………………………………………………………………………………………. 16

3. Methods…………………………………………………………………………………………………………………. 17

3.1 Patient Recruitment and Consent………………………………………………………………………. 17

3.2 Data Collection…………………………………………………………………………………………………… 17

4. Expected Outcomes and Significance………………………………………………………………………. 19

5. Potential Risks…………………………………………………………………………………………………………. 19

6. Safety Reporting………………………………………………………………………………………………………. 20

6.1 Adverse Events…………………………………………………………………………………………………… 20

6.2 Follow-up of Adverse Events………………………………………………………………………………. 20

7. Ethical Considerations……………………………………………………………………………………………… 20

7.1 Regulation Statement…………………………………………………………………………………………. 20

8. Amendment……………………………………………………………………………………………………………… 21

9. References……………………………………………………………………………………………………………….. 22

**List of Abbreviations**

| **ARV** | **Actual resection volume** |
| --- | --- |
| **ARR** | **Actual resection ratio** |
| **DB** | **Blue Dye** |
| **BCS** | **Breast Conserving surgery** |
| **CDCG** | **Clavien Dindo Complication Grade** |
| **CCI** | **Comprehensive Complication Index** |
| **CRF** | **Case Report Form** |
| **DCIS** | **Ductal Cancer In Situ** |
| **FNA** | **Fine Needle Aspiration** |
| **MRI** | **Magnetic Resonance Imaging** |
| **ORV** | **Optimal Resection Volume** |
| **PREMs** | **Patient Reported Experience Measures** |
| **PROs** | **Patient Reported Outcomes** |
| **QoL** | **Quality of Life** |
| **RCT** | **Randomized Controlled Trial** |
| **RFID** | **Radiofrequency identification** |
| **RI** | **Radioisotope** |
| **RSL** | **Radioactive seed localization** |
| **SLN** | **Sentinel Lymph Node** |
| **SLND** | **Sentinel Lymph Node Dissection** |
| **SPIO** | **Superparamagnetic iron oxide nanoparticles** |
| **VAB** | **Vacuum Assisted Biopsy** |

|  |  |
| --- | --- |
| **Summary** |  |
| Title | Magnetic marker or guidewire localization of non-palpable breast cancer in combination with magnetic sentinel lymph node dissection. An open- label, phase 3, pragmati,crandomizedd controlled trial. |
| Clinical Relevance/Background | Preoperative tumour localisation for non- palpable breast cancer lesions has been traditionally performed with a Guidewire. This method is widely available, inexpensive and most of the radiologists and surgeons are familiar with it. However, drawbacks of this technique, including risk of migration, patient discomfort and logistical challenges have led to the development of alternative localisation methods, including magnetic seeds. Previous large cohorts have showed favourable outcomes of magnetic seed localisation, however this has not been investigated in a randomized trial. Furthermore the combination of a totally magnetic technique for lesion localisation and sentinel lymph node detection has not been examined. |
| Objectives | Primary:   - To examine how a magnetic marker compares to the guidewire in successful lesion localisation, minimal excess tissue removal and negative margins in surgery of non-palpable breast lesions. - To evaluate the combination of a ferromagnetic marker for tumour localisation with Superparamagnetic Iron Oxide Nanoparticle (SPIO) for sentinel lymph node detection (SLND) in early breast cancer treatment.   Secondary:   - To evaluate operative times and logistical efficacy of magnetic markers compared to guidewire in the above setting. - To evaluate patient as well as physician satisfaction with the use of this totally magnetic technique. - To evaluate patient quality of life (QoL) |
| Study Design and Methods | Multicentre Pragmatic Randomized Controlled Trial: Patients with confirmed non-palpable ductal cancer in situ (DCIS) or invasive breast cancer, planned for breast conserving surgery (BCS) and SLND will be randomized in two arms.  Arm 1: Lesion localisation with magnetic marker  Arm 2: Lesion localisation with guidewire  Axillary mapping will be done in both arms with the use of SPIO (1-1.5ml Magtrace ©, Endomag, Cambridge,UK) . In the interest of a pragmatic trial, lesion localisation with a magnetic marker as well as SPIO injection will be performed by either a breast surgeron or a radiologist and within a 14 day window before the operation according to local routines or case-per-case convenience. SPIO will be administered peritumoraly. Guidewire localisation will always be performed by a breast radiologist on the day or at most one day before surgery. All cases of localisation will be performed under local anesthetic.  The performing surgeon will document all relevant data in a case reporting form.  All patients will be followed up for postoperative complications, need for re-excision and QoL assessment for 2 years. QoL assessment will be done via PROs questionnaires and the patients experience will be evaluated with PREMs questionnaires.  All involved disciplines (surgeons, radiologists, co-ordinators) will rate their experience with each technique with Likert scale items. |
| Primary Endpoints | To examine the equivalence of magnetic marker and guidewire in successful localisation of non-palpable breast lesions by comparing:   - Re-excision rate - Resection ratio - Sentinel node detection rate |
| Secondary Endpoints | - Adverse events - Operative time - PROs and QoL evaluation - PREMs and cost- effectives analysis |
| Statistical Analysis Plan | A pragmatic randomized open label study to investigate the equivalence of a magnetic marker localisation to guidewire localisation of non-palpable breast lesions. |
| Patient Population | Patients with biopsy confirmed non-palpable breast malignancies planned for BCS and SLND. |
| Study Duration | 5 years |
| Inclusion Criteria | -Patients aged ≥ 18 years  -Patients with ductal cancer in situ (DCIS) or invasive breast cancer (T1-T3) requiring  localisation planned for primary surgery including sentinel node  biopsy. |
| Exclusion Criteria | -Intolerance / hypersensitivity to iron or dextran compounds or  Sienna XP  -Patients with iron-overload disease.  -Patients with pacemakers or other implantable devices in the  chest-wall, or prosthesis in the shoulder.  -Patients deprived of liberty or under guardianship.  -Pregnant or lactating patients.  -Inability to provide informed consent. |

**1.Background**

**1.1 Surgical treatment of Non-palpable Breast Cancer**

Breast cancer is the most commonly diagnosed noncutaneous malignancy in women. Its incidence has increased by an average of 0.4% each year, according to the Surveillance, Epidemiology, and End Results Program (SEER) database^1^. The refinement and wide implementation of screening programs as well as improvements in breast imaging have led to increased diagnosis of breast cancer at a pre-symptomatic stage, that is, non-palpable tumors and clinically negative axilla^2^. Breast-conserving surgery (BCS) is increasingly preferred by this patient group, given that when combined with radiotherapy, it confers equal oncological outcomes to mastectomy while ensuring better QoL ^3–5^. Simultaneously, axillary management has evolved from routine axillary clearance to sentinel node dissection (SLND), which is currently the standard treatment for clinically negative axilla^6^.

**1.2 Preoperative Lesion Localization**

The principle of breast-conserving surgery is twofold and comprises tumor excision with adequate margins, whereas healthy breast parenchyma is spared to ensure optimal cosmetic and functional outcomes. Accurate preoperative lesion localization is essential for achieving this.

**1.2.1 Guidewire Localization**

Since its introduction in the late 1970s, the guidewire has been the standard of care for breast lesion localisation ^7^. It is an inexpensive and widely available method with which most radiologists and surgeons are familiar.

However, various complications have been reported, including wire transection and retention of wire fragments in the breast or migration with damage to the surrounding structures ^8–10^. Furthermore, patient discomfort and pain render this method less appealing.

One of the most apparent disadvantages of this technique is the logistical challenge that it poses in theatre planning. A guidewire has to be inserted on the day of the surgery, which makes operation planning rather copious and may cause delays that not only affect the workload of radiologists and surgeons, but also pose a financial burden on the healthcare system.

**1.2.2 Non-Wire Localization Methods**

The aforementioned drawbacks of the guidewire technique have led to the development of novel methods that primarily aim to detach the preoperative localization from the operation day.

One of the first non-wire markers introduced in the early 2000s was radioactive seed localization (RSL), which uses ^125^I seeds for lesion localization, with many studies confirming favorable outcomes for guidewire localization^11,12^.

The strict regulations and technical difficulties that surround the use of radioactive seeds led to the development of non-radioactive markers such as radiofrequency (RFID) tags ^13,14^, radar reflectors ^15,16^ and magnetic seeds ^17–19^.

All wireless methods, despite the technology that they employ, are based on decoupling lesion localisation from surgery and facilitate theatre logistics.

**1.2.3 Ferromagnetic Seeds**

The Magseed © (Endomag, Cambridge, UK) magnetic marker system comprises a ferromagnetic marker and an 18 gauge needle delivery system used to deliver the marker to the intended deployment location. The stainless-steel marker is magnetically detectable but does not magnetically attract other metallic objects. The product was designed as a single-use device that is sterile in an individually sealed tyvek pouch. The Magseed© magnetic marker is 5mm long and has a diameter of 0.9mm. It is deployed under either ultrasound (US) or mammography guidance. The needle delivery system and markers are visible under both modalities. Magseed© is not suitable for MRI-guided deployment because the needle delivery system is not MRI-compatible. Using the delivery system, the marker is placed percutaneously in the breast for up to 30 days before surgery. A postplacement mammogram can be used to confirm that the marker is in the desired position in the breast.

During surgery, the marker facilitates magnetic localization of the target lesion using a SentiMag© (Endomag, Cambridge,UK) probe. Initially, the probe facilitated the identification of the approximate location of the marker before incision placement. After incision and within the surgical site, the probe was used to detect and localize the lesion for excision. For some lesions, the position of the incision can be determined by other means such as a skin mark.

The SentiMag© probe generates an alternating magnetic field that temporarily magnetizes the marker. The magnetic signature generated by the marker is then detected by a sensitive magnetometer in the probe. The unit displays a numerical reading and emits an audible tone that increases in frequency (pitch) with the marker's proximity to the probe. Once the marker has been localized, it is excised with the lesion.


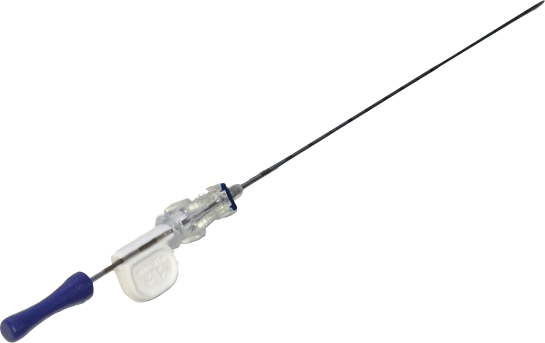

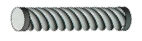

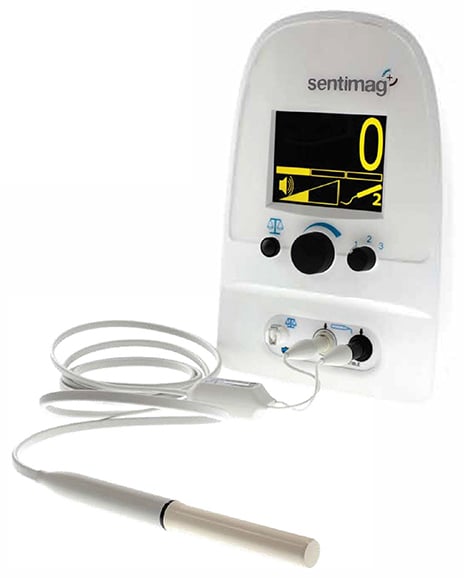


Needle delivery System Ferromagnetic Seed Sentimag© probe

**1.3 Sentinel Lymph Node Detection with Superparamagnetic iron oxide nanoparticles**

Traditionally, axillary mapping and sentinel lymph node (SLN) detection have been performed using radioisotope (RI) with or without blue dye (BD)^20^. Although highly reliable, this combination poses challenges due to restricted access to nuclear medicine facilities and strict regulations, whereas the short half-life of RI and the allergenic reactions of BD limit its administration on the day of surgery, complicating logistics.

Superparamagnetic iron oxide nanoparticles (SPIO) are iron oxide nanoparticles covered in an organic coating that become transiently magnetic in the vicinity of a magnetic source. Magtrace ^©^ (Endomag, Cambridge, UK) is a liquid suspension of carboxydextran-coated iron oxide nanoparticles in injectable water. Each milliliter of Magtrace ^©^ contains approximately 28 mg of iron. SPIO has shown comparable performance to RI ± BD with the additional advantage of a wider timeframe for preoperative administration^21–23^. The perceived drawbacks of this method include skin staining and artifacts on postoperative magnetic resonance imaging (MRI) ^24^.

**1.4 The “MAGnetic marker To deTect primary Lesion and sentinel node in breast cancer” (MAGtotal) concept.**

The combined technique involves seed placement on the ventral surface of the tumor, while SPIO is injected dorsally to the tumor between the tumor and the chest-wall. Thus, we can achieve an enhanced signal that facilitates tumor localization, even in deeper lesions and larger breasts. Simultaneously, this technique is expected to minimize the risk of intraoperative confusion due to overlapping signals, which could lead to excision of larger specimens. Another concern that is addressed when SPIO is administered dorsally to the tumor is that the bulk of the brown-colored suspension is concentrated deep in the breast tissue and between the tumor and pectoral fascia, minimizing the risk of skin staining. The MAGtotal technique was tested in a pilot study with 32 patients with encouraging results that led to the design of a phase 3 RCT (100% clear margins, 1.49 resection ratio, 100% SLN detection)^25^.

**The Magtotal concept**

**
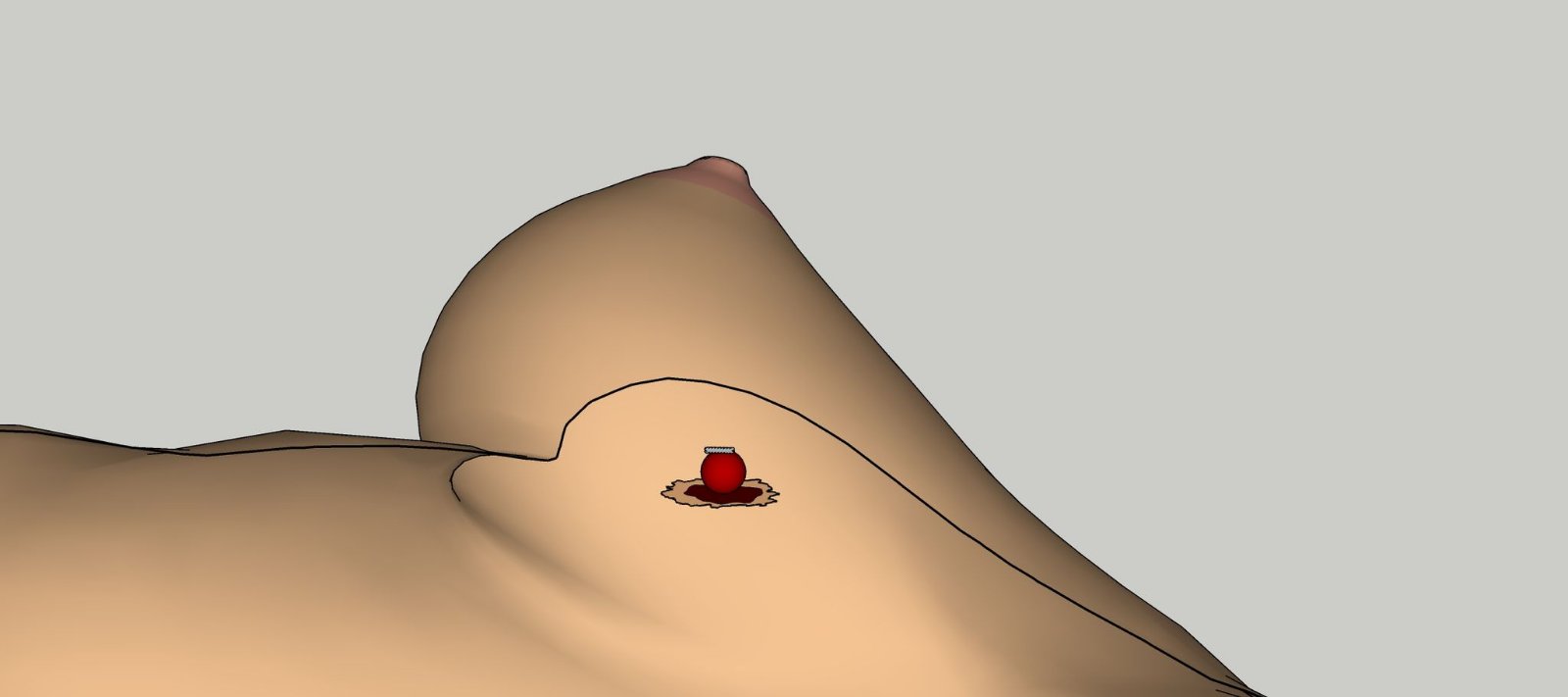
**

**2.Investigational Plan**

**2.1 Title**

Magnetic marker or guidewire localization of non-palpable breast cancer in combination with magnetic sentinel lymph node dissection. An open-label, phase 3, pragmatic, randomized controlled trial.

**2.2 Objectives**

**2.2.1 Primary Objective**

1. The primary objective is a composite of the three components of successful breast conserving surgery for early breast cancer. These components include the re-excision rate, minimal excess tissue removal, and successful SLN detection. The aim of this trial is to compare these three variables between the two arms.

The hypotheses to be tested are:

1. Magnetic marker lesion localization is not equivalent to guidewire localization in terms of re-excision rates due to inadequate surgical margins. Adequate margins are considered “no tumour on ink” for invasive cancer and 2 mm for pure DCIS and will be assessed by specialized breast pathologists.

2. Magnetic marker lesion localization is not equivalent to guidewire localization in terms of excess tissue removal. This will be expressed as the resection ratio and will be assessed by the ratio of the Optimal Resection Ratio (ORV) to the Actual Resection Ratio (ARV).

- ORV will be calculated for each tumour based on the radiological dimensions of the tumor (in cases of discordance between different modalities, the largest dimension will be used) using the volume calculation formula for ellipsoid tumours
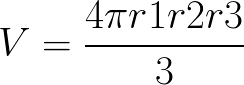
, where *r*1= x radius +1cm, *r*2=y radius+1cm, *r*3= y radius +1cm. In all dimensions, 1 cm is added as it is the universally accepted macroscopic margin for breast lesions^26^.

ARV will be calculated based on the specimen weight.

Optimally, the ratio $\frac{ARV}{ORV}$ should be as close to 1 as possible to ensure that no unnecessary tissue is removed which may jeopardize the cosmetic and functional outcomes of a breast conserving operation.

3. The combination of magnetic markers and SPIO is not equivalent to the combination of a guidewire and SPIO for SLN detection.

**2.2.2. Secondary Objectives**

1. Evaluation of the frequency of adverse events, that is, failed localization and postoperative complications, with each method.

2. Time to specimen and total operative time for each technique.

3. The evaluation of healthcare personal experience with each technique via Likert scale items

4. Evaluation of patients’ QoL through PROs and their experience with each technique via PREMs questionnaires.

5. Cost-effectiveness analysis for each technique.

**2.3 Patient Population and Indications**

**2.3.1 Inclusion Criteria**

1. Patients aged ≥ 18 years

2. Patients with DCIS or invasive breast cancer (T1-T3) requiring localisation planned for primary surgery including sentinel node biopsy

**2.3.2 Exclusion Criteria**

1. Intolerance/hypersensitivity to iron or dextran compounds.

2. Patients with iron-overload disease.

3. Patients with pacemakers or other implantable devices in the chest wall or prosthesis of the shoulder.

4. Patients deprived of liberty or under guardianship.

5. Pregnant or lactating patients.

6. Inability to provide informed consent.

**2.4 Study Design**

The study is designed as a randomised controlled trial that complies with the principles of pragmatism. The extent of pragmatism was quantified using the PRagmatic-Explanatory Continuum Indicator Summary 2 (PRECIS-2) score, a tool used to facilitate the design of pragmatic trials^27^.PRECIS-2 evaluates the applicability of a trial within 10 domains and produces a PRECIS-2 wheel, as shown in the table below.

**Table:** **PRECIS-2 scores for trial domains**

| **Domain** | **Score** | **Rationale** |
| --- | --- | --- |
| Eligiblity criteria | 5 | Patients with breast tumours that need localisation- Exclusion criteria related to reaction to the material and not the delivery of the technique. |
| Recruitment Path | 5 | Recruitment performed during routine clinic appointment for surgery planning. |
| Setting | 4 | Study conducted in two university and one county hospital, with various case volumes and level of experience. However, all three centres are in the same country. |
| Organisation intervention | 5 | No prior training or additional resources needed for and of the disciplines involved in the trial. |
| Flexibility-delivery | 5 | No monitor or specific advice on co-intervention. |
| Flexibility -adherence | 5 | No special adherence measures. |
| Follow up | 4 | A 2 year follow up which is not routine in usual care and QoL measurements. |
| Outcome | 5 | Primary outcomes are directly relevant to participants |
| Analysis | 5 | All available data used |

PRECIS-2 wheel

Patients with non-palpable DCIS or invasive breast cancer confirmed by fine-needle aspiration (FNA), core biopsy, or vacuum-assisted biopsy (VAB), which are planned for BCS and SLND, will be asked to participate in the trial. Upon their consent, they will be randomized to either arm 1 (lesion localization with magnetic marker) or arm 2 (lesion localization with guidewire).

**2.5 Localisation Technique**

The magnetic marker that will be used is Magseed© (Endomag, Cambridge,UK). All patients will receive peritumoral SPIO (1-1.5 ml Magtrace©) up to 14 days before surgery. SPIO administration and magnetic marker insertion may be performed by either a radiologist or breast surgeon, but always with radiological guidance. SPIO will be injected in the dorsal surface of the tumor while the magnetic marker will be placed on the ventral surface of the tumour. Guidewire placement will be performed exclusively by a breast radiologist as per routine, with the tip of the guidewire advancing approximately 1 cm past the lesion to ensure adequate macroscopic margins.

All specimens will be sent to the radiology department after excision and orientation to ensure that the tumor and marker are within the specimen. The radiological margins will be documented.

The trial is open to all breast radiologists and surgeons regardless of their level of experience or familiarity with the technique to ensure that the results are applicable to any clinical setting.

**2.6 Sampling and Data Analysis Plan**

The sample size is calculated based on the principle of equivalence, allowing for the detection of a significant difference in resection ratios of 0.3 adjusted for a non-inferiority margin of 4% for re-excision rates and SLN detection. The literature suggests a resection ratio for the guidewire between 2-2.8. To obtain robust results, we lowered that to 1.8 and accepted a 1.5 resection ratio for the totally magnetic technique stemming from our previous pilot study ^25^. Two-sided p-value is set at 0.05 and power to 80%. An additional 10% to sample size calculation will be recruited as inflation to pragmatic settings and tolerance.

The participants will be randomized with an allocation ratio of 1:1 in blocks of 8. The R-Randomizer package will be used for the randomization. The allocated arms will be in dark envelopes, and neither of the involved disciplines will be able to know the arm in which the participants will be randomized beforehand. All participants will receive a code upon randomization consisting of the first three letters of the recruiting center and a number that will be consecutive. The data will be collected by appointed research coordinators.

**2.7 Statistical Analysis**

Descriptive statistics will be performed. Continuous variables will be controlled for normality by means of the Kolmogorov Smirnoff and Shapiro- Wilk tests. For normally distributed data, means and standard deviation (SD) will be calculated whereas median with interquartile range (iqr) or range will be used to summarize variables without normal distribution. Detection of significance between groups for these variables will be performed using the Student´s t-test or the Mann-Whitney test**.**

**3.Methods**

**3.1 Patient Recruitment and Consent**

Patients will be informed about the study during their first visit to the outpatient breast clinic after being informed about their diagnosis and surgical plan. Given the delicate nature of the diagnosis, adequate time will be given to the patients to process the information and when indicated, a second visit will be planned to give them more information about their diagnosis, the proposed treatment plan, and, eventually, their participation in the study. All patients who are interested in the study will be given a patient information form that they can keep and a patient consent form in which they can opt for being sent a copy of the published data of the study. Patients may decide to withdraw from the study at any point before localization is performed.

**3.2 Data collection**

The data collected included baseline patient and tumor characteristics, intraoperative details, and postoperative data including the pathology report, report of complications, and SPIO-induced skin staining.

Preoperative data:

- Age, Sex, BMI
- Radiographic tumour size and BIRADS (both mammogram and Ultrasound)
- Optimal resection volume
- Tumour location (side, quadrant, distance from nipple areola complex)
- Clinical size
- Preoperative core biopsy, needle size
- Histology, grade and receptor status
- Date of SPIO injection, US guided or not and volume
- Date of tumor localization, method employed, radiological guidance, time, and complications of the procedure.

Intraoperative data:

- Date of primary surgery
- Type of procedure
- Sentimag counts in breast and axilla after induction of anaesthesia
- Time from skin incision to specimen excision
- Radiologic margins (min, max)
- Skin excision information
- Sentimag counts on specimen and background
- Specimen weight
- Tracer or tracers that are used
- Successful SPIO SLNB
- Successful SLNB by any method
- Number of SLN
- Axillary procedure

Postoperatice data

- Date of pathology report
- Date answer was delivered to patient
- Specimen dimensions
- Histology and grade
- Size
- Receptor status
- Margin status and closest margin
- SLN number, number of metastases if any in SLN
- Skin staining and size
- Residual magnetic signal
- Planned reoperation
- Breast specific complications within 30 days, CD and CCI grading
- BREAST-Q preoperative, expectations, immediate postoperative, 6 months postoperative, 12 months postoperative

**4. Expected Outcomes and Significance**

The use of a magnetic technique in nonpalpable breast lesions is expected to facilitate operating logistics without compromising oncological and cosmetic outcomes. The use of magnetic seeds and SPIO allows lesion localization and axillary mapping within a much wider timeframe, which may result in reduced theater costs, more efficient planning, and better working conditions for surgeons and radiologists. It is also expected that patient comfort and satisfaction will increase as they will not have to tolerate a wire protruding from their breast and the stress of having everything done on the operation day.

**5. Potential Risks**

Magnetic seeds and SPIO are nonradioactive and nonallergenic substances. No special protocols must be followed for use or disposal.

Concerns have been raised about the risk of skin discoloration which may happen in certain cases after SPIO injection. While this is not harmful, but it is aesthetically displeasing and may persist for months before it fades. The possibility of skin staining should be discussed with each patient before administration and all steps will be taken to avoid it. According to a recent meta-analysis, skin staining may be contained with smaller doses and peritumoural administration, a practice that will be followed in this trial^28^.

Another area of concern is the incompatibility of Magseed© and SPIO to Magnetic Resonance Imaging (MRI). Non-clinical testing has demonstrated that the marker can be scanned safely in an MRI under the following conditions:

• Static magnetic field of 1.5-Tesla (1.5 T) or 3-Tesla (3 T).

• Maximum spatial field gradient of 4,000 G/cm (40 T/m).

• Maximum MR system reported whole body averaged specific absorption rate (SAR) of 4 W/kg (First Level Controlled Operating Mode).

However, it creates artifacts that affect the diagnostic efficacy of MRI, which is why localization must be done after all diagnostic imaging is completed. SPIO also affects MRI diagnostic accuracy, and the traces left behind during the operation affect postoperative imaging as well. Smaller doses and injection dorsally to the tumour with the intent of removing the whole area along with the tumour are measures that will be taken during this trial to reduce this phenomenon.

**6. Safety Reporting**

**6.1 Adverse events**

An adverse event refers to any negative or unintended medical occurrence that is associated with medical intervention, including adverse drug reactions, treatment-related symptoms or diseases, or other adverse experiences that happen during the course of a clinical trial. These events can be mild, serious, or life-threatening, and can range from minor side effects to serious injury or death.

All adverse events will be reported and documented to the principal investigator and appropriate measures will be applied depending on the severity of the adverse effects.

**6.2 Follow-up of adverse events**

All adverse events will be closely monitored until they are resolved or stabilized. Appropriate actions will be taken in case an adverse event requires further medical care or examinations.

**7. Ethical Considerations**

**7.1 Regulation Statement**

Participants information will be protected under the Personal Data Act. The study will be conducted according to the Declaration of Helsinki (64th WMA General Assembly, Fortaleza, Brazil, October 2013) and the Act on Patient Insurance in Sweden. There will be no financial compensation for study participants or study sites.

**8. Amendment**

Date: April 2020

Purpose: To modify the method of localization for breast lesions in response to the COVID-19 pandemic, in order to prevent delays in planned surgery.

Background: In light of the current COVID-19 pandemic, [Investigator/Sponsor name] has determined that it is in the best interest of the trial participants to allow for increased flexibility in the method of preoperative localization for breast lesions.

Amendment: Effective immediately, trial participants and/or personnel, involved in surgery planning will be allowed to choose the method of localization (guidewire or magseed) that is most convenient for their case and ensures that there will be no delays in their planned operation, regardless of the randomization assignment.

Implementation: The change in method of localization will be documented in the case report form and recorded in the trial database. The randomization assignment will still be recorded, but the choice of localization method will take precedence.

Rationale: This amendment is being made to minimize the burden on trial participants during the COVID-19 pandemic and to ensure their comfort and convenience.

Approval: This amendment has been reviewed and approved by the Swedish Ethics Committee

Signed: Andreas Karakatsanis

**References**

1. Cronin KA, Lake AJ, Scott S, et al. Annual Report to the Nation on the Status of Cancer, part I: National cancer statistics. *Cancer*. 2018;124(13):2785-2800. doi:10.1002/cncr.31551

2. Cady B, Stone MD, Schuler JG, Thakur R, Wanner MA, Lavin PT. The New Era in Breast Cancer: Invasion, Size, and Nodal Involvement Dramatically Decreasing as a Result of Mammographic Screening. *Arch Surg*. 1996;131(3):301-308. doi:10.1001/archsurg.1996.01430150079015

3. Fisher B, Anderson S, Bryant J, et al. Twenty-Year Follow-up of a Randomized Trial Comparing Total Mastectomy, Lumpectomy, and Lumpectomy plus Irradiation for the Treatment of Invasive Breast Cancer. *N Engl J Med*. 2002;347(16):1233-1241. doi:10.1056/NEJMoa022152

4. Veronesi U, Viale G, Paganelli G, et al. Sentinel lymph node biopsy in breast cancer: ten-year results of a randomized controlled study. *Ann Surg*. 2010;251(4):595-600. doi:10.1097/SLA.0b013e3181c0e92a

5. Engel J, Kerr J, Schlesinger-Raab A, Sauer H, Hölzel D. Quality of life following breast-conserving therapy or mastectomy: results of a 5-year prospective study. *Breast J*. 2004;10(3):223-231. doi:10.1111/j.1075-122X.2004.21323.x

6. Lyman GH, Giuliano AE, Somerfield MR, et al. American Society of Clinical Oncology Guideline Recommendations for Sentinel Lymph Node Biopsy in Early-Stage Breast Cancer. *J Clin Oncol*. 2005;23(30):7703-7720. doi:10.1200/JCO.2005.08.001

7. Hall FM, Kopans DB, Sadowsky NL, Homer MJ. Development of Wire Localization for Occult Breast Lesions: Boston Remembrances. *Radiology*. 2013;268(3):622-627. doi:10.1148/radiol.13121943

8. Davis P, Wechsler R, Feig S, March D. Migration of breast biopsy localization wire. *Am J Roentgenol*. 1988;150(4):787-788. doi:10.2214/ajr.150.4.787

9. Banitalebi H, Skaane P. Migration of the breast biopsy localization wire to the pulmonary hilus. *Acta Radiol*. 2005;46(1):28-31. doi:10.1080/02841850510015956

10. Seifi A, Axelrod H, Nascimento T, et al. Migration of Guidewire After Surgical Breast Biopsy: An Unusual Case Report. *Cardiovasc Intervent Radiol*. 2009;32(5):1087-1090. doi:10.1007/s00270-009-9620-9

11. Gray RJ, Salud C, Nguyen K, et al. Randomized prospective evaluation of a novel technique for biopsy or lumpectomy of nonpalpable breast lesions: radioactive seed versus wire localization. *Ann Surg Oncol*. 2001;8(9):711-715. doi:10.1007/s10434-001-0711-3

12. Alderliesten T, Loo CE, Pengel KE, Rutgers EJT, Gilhuijs KGA, Vrancken Peeters MJTFD. Radioactive seed localization of breast lesions: an adequate localization method without seed migration. *Breast J*. 2011;17(6):594-601. doi:10.1111/j.1524-4741.2011.01155.x

13. Reicher JJ, Reicher MA, Thomas M, Petcavich R. Radiofrequency identification tags for preoperative tumor localization: proof of concept. *AJR Am J Roentgenol*. 2008;191(5):1359-1365. doi:10.2214/AJR.08.1023

14. Dauphine C, Reicher JJ, Reicher MA, Gondusky C, Khalkhali I, Kim M. A prospective clinical study to evaluate the safety and performance of wireless localization of nonpalpable breast lesions using radiofrequency identification technology. *AJR Am J Roentgenol*. 2015;204(6):W720-723. doi:10.2214/AJR.14.13201

15. Cox CE, Garcia-Henriquez N, Glancy MJ, et al. Pilot Study of a New Nonradioactive Surgical Guidance Technology for Locating Nonpalpable Breast Lesions. *Ann Surg Oncol*. 2016;23(6):1824-1830. doi:10.1245/s10434-015-5079-x

16. Cox CE, Russell S, Prowler V, et al. A Prospective, Single Arm, Multi-site, Clinical Evaluation of a Nonradioactive Surgical Guidance Technology for the Location of Nonpalpable Breast Lesions during Excision. *Ann Surg Oncol*. 2016;23(10):3168-3174. doi:10.1245/s10434-016-5405-y

17. Hayes MK. Update on Preoperative Breast Localization. *Radiol Clin North Am*. 2017;55(3):591-603. doi:10.1016/j.rcl.2016.12.012

18. Price ER, Khoury AL, Esserman LJ, Joe BN, Alvarado MD. Initial Clinical Experience With an Inducible Magnetic Seed System for Preoperative Breast Lesion Localization. *AJR Am J Roentgenol*. 2018;210(4):913-917. doi:10.2214/AJR.17.18345

19. Harvey JR, Lim Y, Murphy J, et al. Safety and feasibility of breast lesion localization using magnetic seeds (Magseed): a multi-centre, open-label cohort study. *Breast Cancer Res Treat*. 2018;169(3):531-536. doi:10.1007/s10549-018-4709-y

20. Hung WK, Chan CM, Ying M, Chong SF, Mak KL, Yip AWC. Randomized clinical trial comparing blue dye with combined dye and isotope for sentinel lymph node biopsy in breast cancer. *Br J Surg*. 2005;92(12):1494-1497. doi:10.1002/bjs.5211

21. Karakatsanis A, Christiansen PM, Fischer L, et al. The Nordic SentiMag trial: a comparison of super paramagnetic iron oxide (SPIO) nanoparticles versus Tc(99) and patent blue in the detection of sentinel node (SN) in patients with breast cancer and a meta-analysis of earlier studies. *Breast Cancer Res Treat*. 2016;157(2):281-294. doi:10.1007/s10549-016-3809-9

22. Thill M, Kurylcio A, Welter R, et al. The Central-European SentiMag study: sentinel lymph node biopsy with superparamagnetic iron oxide (SPIO) vs. radioisotope. *Breast Edinb Scotl*. 2014;23(2):175-179. doi:10.1016/j.breast.2014.01.004

23. Rubio IT, Diaz-Botero S, Esgueva A, et al. The superparamagnetic iron oxide is equivalent to the Tc99 radiotracer method for identifying the sentinel lymph node in breast cancer. *Eur J Surg Oncol J Eur Soc Surg Oncol Br Assoc Surg Oncol*. 2015;41(1):46-51. doi:10.1016/j.ejso.2014.11.006

24. Krischer B, Forte S, Niemann T, Kubik-Huch RA, Leo C. Feasibility of breast MRI after sentinel procedure for breast cancer with superparamagnetic tracers. *Eur J Surg Oncol J Eur Soc Surg Oncol Br Assoc Surg Oncol*. 2018;44(1):74-79. doi:10.1016/j.ejso.2017.11.016

25. Hersi AF, Eriksson S, Ramos J, Abdsaleh S, Wärnberg F, Karakatsanis A. A combined, totally magnetic technique with a magnetic marker for non-palpable tumour localization and superparamagnetic iron oxide nanoparticles for sentinel lymph node detection in breast cancer surgery. *Eur J Surg Oncol J Eur Soc Surg Oncol Br Assoc Surg Oncol*. 2019;45(4):544-549. doi:10.1016/j.ejso.2018.10.064

26. Krekel NMA, Zonderhuis BM, Stockmann HBAC, et al. A comparison of three methods for nonpalpable breast cancer excision. *Eur J Surg Oncol EJSO*. 2011;37(2):109-115. doi:10.1016/j.ejso.2010.12.006

27. Loudon K, Treweek S, Sullivan F, Donnan P, Thorpe KE, Zwarenstein M. The PRECIS-2 tool: designing trials that are fit for purpose. *BMJ*. 2015;350:h2147. doi:10.1136/bmj.h2147

28. Pantiora E, Tasoulis MK, Valachis A, et al. Evolution and refinement of magnetically guided sentinel lymph node detection in breast cancer: meta-analysis. *Br J Surg*. Published online December 23, 2022:znac426. doi:10.1093/bjs/znac426
