# Supplementary material for: Magnetic seed versus guidewire-based breast cancer localization with magnetic lymph node detection: cost-minimization analysis
Source: Br J Surg. 2025 Dec 1;112(12):znaf253. doi: 10.1093/bjs/znaf253 (PMC12667277; doi:10.1093/bjs/znaf253)
Supplement: znaf253_Supplementary_Data [file znaf253_supplementary_data.zip › Supplementary_material.docx]

Magnetic seed vs guidewire breast cancer localization with magnetic lymph node detection: a cost-minimisation analysis

Pantiora E^1,2^, Sampaio F^3^, Jazrawi A^4,5^, Wärnberg F^1,2,6,7^, Eriksson S^4,5^, Karakatsanis A^1,2^.

1. Department of Surgical Sciences, Uppsala University, Uppsala, Sweden
2. Section for Breast Surgery, Department of Surgery, Uppsala University Hospital, Uppsala, Sweden
3. Department of Public Health and Caring Sciences, Uppsala University, Uppsala, Sweden
4. Center for Clinical Research, Department of Surgical Sciences,Uppsala University, Västerås, Sweden
5. Section for Breast Surgery, Department of Surgery, Västmanlands County Hospital, Västerås, Sweden
6. Sahlgrenska Center for Cancer Research, Department of Surgery, Institute of Clinical Sciences, Sahlgrenska Academy, University of Gothenburg, Gothenburg, Sweden
7. Department of Surgery, Sahlgrenska University, Hospital, Gothenburg, Sweden

Supplementary Material.

Table S1: Unadjusted analysis for the main trial and the sensitivity analyses.

Table S2: Unadjusted and adjusted sensitivity analysis. Sensitivity analysis 1 denotes all magnetic seeds placed by radiologists. Sensitivity analysis 2 denotes all magnetic seeds placed by surgeons, along with SPIO administration, for all tumours detectable on ultrasound.

Supplement: CHEERS 2022 Checklist

Table S1: Unadjusted analysis for the main trial and the sensitivity analyses.

| **A: Type of device** | | | | | |
| --- | --- | --- | --- | --- | --- |
|  | Guidewire | Magnetic Marker |  | Difference (95%) CI | p-value |
| ***1. In-trial results*** |  |  |  |  |  |
| Mean (SD) | 3337 (1350) | 3274 (1105) |  | -63 (-302, 174) | 0.599* |
| Mean (95 % CI) | 3337 (3151, 3524) | 3274 (3124, 3160) |  |  |  |
| Median (IQR) | 3034 (2663, 3696) | 3031 (2518, 3696) |  |  | 0.886** |
| Bootstrapped Mean (95% CI) | 3337 (3157, 3527) | 3274 (3127, 3423) |  | -63 (-303, 173) | 0.596* |
| Bootstrapped Median (95% CI) | 3043 (2857, 3175) | 3031 (2909, 3227) |  |  |  |
| ***2. Sensitivity Analysis 1*** |  |  |  |  |  |
| Mean (SD) | 3620 (1350) | 3556 (1105) |  | -63 (-302, 175) | 0.601* |
| Mean (95 % CI) | 3620 (3433, 3806) | 3556 (3406, 3706) |  |  |  |
| Median (IQR) | 3316 (2663, 4340) | 3313 (2800, 3978) |  |  | 0.886** |
| Bootstrapped Mean (95% CI) | 3620 (3439, 3810) | 3556 (3410, 3705) |  | -63 (-303, 173) | 0.596* |
| Bootstrapped Median (95% CI) | 3316 (3139, 3457) | 3313 (3191, 3509) |  |  |  |
| ***3. Sensitivity Analysis 2*** |  |  |  |  |  |
| Mean (SD) | 3618 (1350) | 3287 (1119) |  | -330 (-570, -90) | 0.007* |
| Mean (95 % CI) | 3618 (3432, 3805) | 3287 (3138, 3439) |  |  |  |
| Median (IQR) | 3297 (2663, 4340) | 3022 (2528, 3694) |  |  | 0.056** |
| Bootstrapped Mean (95% CI) | 3618 (3438, 3810) | 3254 (3140, 3296) |  | -330 (-571, -95) | 0.011* |
| Bootstrapped Median (95% CI) | 3297 (3101, 3457) | 3022 (2903, 3296) |  |  |  |
|  |  |  |  |  |  |
| **B: Type of surgery** | | | | | |
|  | WLE | OPBCS | TM | Difference (95%) CI |  |
| ***1. In-trial results*** |  |  |  |  |  |
| Mean (SD) | 3126 (1087) | 3730 (1284) | 5232 (1475) | 604 (144, 1064) // 2106 (1280, 2932) | <0.001* |
| Mean (95 % CI) | 3126 (3010, 3241) | 3722 (3365, 4078) | 5232 (4560, 5903) |  |  |
| Median (IQR) | 2934 (2359, 3583) | 3266 (2751, 4371) | 4848 (4168, 5710) |  | <0.001** |
| Bootstrapped Mean (95% CI) | 3126 (3020, 3240) | 3730 (3383, 4104) | 5232 (4639, 5863) | 604 (251, 1006) // 2106 (1481, 2817) | <0.001* |
| Bootstrapped Median (95% CI) | 2934 (2828, 3031) | 3266 (3053, 3942) | 4848 (4269, 5546) |  |  |
| ***2. Sensitivity Analysis 1*** |  |  |  |  |  |
| Mean (SD) | 3408 (1087) | 4013 (1284) | 5515 (1475) | 604 (144, 1065) // 2106 (1280, 2933) | <0.001* |
| Mean (95 % CI) | 3408 (3293, 3524) | 4012 (3648, 4378) | 5515 (4843, 6187) |  |  |
| Median (IQR) | 3216 (2641, 3865) | 3548 (3033, 4653) | 5131 (4455, 5993) |  | <0.001** |
| Bootstrapped Mean (95% CI) | 3408 (3302, 3523) | 4012 (3666, 4387) | 5515 (4922, 6146) | 604 (251, 1006) // 2106 (1481, 2818) | <0.001* |
| Bootstrapped Median (95% CI) | 3216 (3112, 3313) | 3548 (3335, 4225) | 5131 (4554, 5828) |  |  |
| ***3. Sensitivity Analysis 2*** |  |  |  |  |  |
| Mean (SD) | 3276 (1105) | 3857 (1326) | 5349 (1521) | 580 (106, 1055) // 2073 (1221, 2925) | <0.001* |
| Mean (95 % CI) | 3276 (3158, 3393) | 3857 (3480, 4234) | 5349 (-4657, -5268) |  |  |
| Median (IQR) | 3022 (-2503, -3732) | (-2942, -4365) | (-4839, -5993) |  | <0.001** |
| Bootstrapped Mean (95% CI) | 3276 (3169, -3390) | 3857 (-3427, -4156) | 5349 (-4732, -5982) | 2073 (1416, 2799) | <0.001* |
| Bootstrapped Median (95% CI) | 3022 (-2902, -3161) | 3435 (-3180, -4009) | 4839 (-4365, -5828) |  |  |
|  |  |  |  |  |  |
| **C: Single localisation session** | | | | | |
|  | No | Yes |  | Difference (95% CI) |  |
| ***1. In-trial results*** |  |  |  |  |  |
| Mean (SD) | 3498 (1230) | 3015 (1180) |  | 481 (243, 720) | <0.001* |
| Mean (95 % CI) | 3498 (3345, 3652) | 3015 (2833, 3196) |  |  |  |
| Median (IQR) | 3237 (2683, 3976) | 2737 (2225, 3543) |  |  | <0.001** |
| Bootstrapped Mean (95% CI) | 3498 (3233, 3527) | 3015 (2857, 3186) |  | 481 (245, 705) | <0.001* |
| Bootstrapped Median (95% CI) | 3237 (3058, 3453) | 2737 (2477, 2869) |  |  |  |
| 2. Sensitivity Analysis 1 |  |  |  |  |  |
| Mean (SD) | 3781 (1230) | 3297 (1180) |  | 481 (243, 720) | <0.001* |
| Mean (95 % CI) | 3781 (3636, 3934) | 3297 (3116, 3479) |  |  |  |
| Median (IQR) | 3520 (2952, 4258) | 3019 (2507, 3825) |  |  | <0.001** |
| Bootstrapped Mean (95% CI) | 3781 (3626, 3931) | 3297 (3130, 3563) |  | 481 (245, 705) | <0.001* |
| Bootstrapped Median (95% CI) | 3520 (3340, 3735) | 3019 (2765, 3152) |  |  |  |
| 3. Sensitivity Analysis 2 |  |  |  |  |  |
| Mean (SD) | 3585 (1277) | 3250 (1181) |  | 332 (88, 576) | 0.008* |
| Mean (95 % CI) | 3585 (3425, 3744) | 3250 (3068, 3431) |  |  |  |
| Median (IQR) | 3337 (2706, 4049) | 2904 (2428, 3694) |  |  | 0.006** |
| Bootstrapped Mean (95% CI) | 3585 (3427, 3739) | 3250 (3084, 3422) |  | 332 (94, 561) | 0.007* |
| Bootstrapped Median (95% CI) | 3337 (3101, 3496) | 2904 (2744, 3139) |  |  |  |

Table S2: Unadjusted and adjusted sensitivity analysis.

|  | Unadjusted analysis | | | Adjusted analysis | | | |
| --- | --- | --- | --- | --- | --- | --- | --- |
|  | Mean (95% CI) | Marginal Difference  (95% CI) | p-value | Coefficient  (95% CI) | Marginal Means  (95% CI) | Difference  (95% CI) | p-value |
| **Sensitivity analysis 1** | | | | | | | |
| Localization device |  |  |  |  |  |  |  |
| Guidewire | 3620 (3433, 3806) | Ref. [0] |  | Ref. [0] | 3798 (3618, 3978) | Ref. [0] |  |
| Seed | 3556 (3406, 3706) | -63 (-302, 175) | 0.601 | -0.110 (-0.178, -0.041) | 3403 (3253, 3554) | -394 (-365, -424) | 0.002 |
|  |  |  |  |  |  |  |  |
| Type of Breast Surgery |  |  |  |  |  |  |  |
| WLE | 3408 (3293, 3524) | Ref. [0] |  | Ref. [0] | 3415 (3307, 3533) | Ref. [0] |  |
| OPBCS | 4012 (3648, 4378) | 604 (144, 1065) |  | 0.144 (0.051, 0.236) | 3948 (3607, 4290) | 528 (301, 756) | 0.002 |
| TM | 5515 (4843, 6187) | 2106 (1280, 2933) | <0.001 | 0.461 (0.322, 0.599) | 5421 (4695, 6147) | 2001 (1388, 2614) | <0.001 |
|  | | | | | | | |
| Single localization session |  |  |  |  |  |  |  |
| Yes | 3297 (3116, 3479) | Ref. [0] |  | Ref. [0] | 3269 (3100, 3439) | Ref. [0] |  |
| No | 3781 (3636, 3934) | 481 (243, 720) | <0.001 | 0.151 (0.081, 0.222) | 3803 (3645, 3960) | 533 (521, 546) | <0.001 |
|  |  |  |  |  |  |  |  |
| **Sensitivity analysis 2** | | | | | | | |
| Localization device |  |  |  |  |  |  |  |
| Guidewire | 3618 (3432, 3805) | Ref. [0] |  | Ref. [0] | 3791 (3611, 3985) | Ref. [0] |  |
| Seed | 3287 (3138, 3439) | -330 (-570, -90) | 0.007 | -0.118 (-0.260, -0.117) | 3145 (3000, 3289) | -653 (-696, -611) | <0.001 |
|  |  |  |  |  |  |  |  |
| Type of Breast Surgery |  |  |  |  |  |  |  |
| WLE | 3276 (3158, 3393) | Ref. [0] |  | Ref. [0] | 3415 (3307, 3533) | Ref. [0] |  |
| OPBCS | 3857 (3480, 4234) | 580 (106, 1055) |  | 0.146 (0.049, 0.242) | 3948 (3607, 4290) | 528 (301, 756) | 0.003 |
| TM | 5349 (-4657, -5268) | 2073 (1221, 2925) | <0.001 | 0.488 (0.344, 0.632) | 5421 (4695, 6147) | 2001 (1388, 2614) | <0.001 |
|  |  |  |  |  |  |  |  |
| Single localization session |  |  |  |  |  |  |  |
| Yes | 3250 (3068, 3431) | Ref. [0] |  | Ref. [0] | 3156 (2988, 3325) | Ref. [0] |  |
| No | 3585 (3425, 3744) | 332 (88, 576) | <0.001 | 0.151 (0.081, 0.222) | 3659 (3498, 3820) | 503 (495, 510) | <0.001 |

Unadjusted and Adjusted Cost Minimisation Sensitivity Analysis. Sensitivity analysis 1 denotes all magnetic seeds placed by radiologists. Sensitivity analysis 2 denotes all magnetic seeds placed by surgeons, along with SPIO administration, for all tumours detectable on ultrasound.

Monetary units are Euros (€). Mean values are presented with 95% CI (confidence intervals). The adjusted analysis is performed with a generalized linear model (gamma family, log link). Ref.: reference category, OPBCS: oncoplastic breast conserving surgery, TM: therapeutic mastopexy/mammaplasty, WLE: wide local excision. *: regression analysis, **: generalised linear regression model.

CHEERS 2022 checklist

| **Topic** | **No.** | **Item** | **Location where item is reported** |
| --- | --- | --- | --- |
| **Title** |  |  |  |
|  | 1 | Identify the study as an economic evaluation and specify the interventions being compared. | Page1, Rows 1-2 |
| **Abstract** |  |  |  |
|  | 2 | Provide a structured summary that highlights context, key methods, results, and alternative analyses. | Page 2 |
| **Introduction** |  |  |  |
| **Background and objectives** | 3 | Give the context for the study, the study question, and its practical relevance for decision making in policy or practice. | Page 3, Rows 64-85 |
| **Methods** |  |  |  |
| **Health economic analysis plan** | 4 | Indicate whether a health economic analysis plan was developed and where available. | Page 3-4, Rows 89-96 |
| **Study population** | 5 | Describe characteristics of the study population (such as age range, demographics, socioeconomic, or clinical characteristics). | Page 4, Rows 99-102 |
| **Setting and location** | 6 | Provide relevant contextual information that may influence findings. | Pages 3-4, Rows 89-96 |
| **Comparators** | 7 | Describe the interventions or strategies being compared and why chosen. | Page 4, Rows 105-107 |
| **Perspective** | 8 | State the perspective(s) adopted by the study and why chosen. | Page 3, Rows 89-90 |
| **Time horizon** | 9 | State the time horizon for the study and why appropriate. | Page 6, Rows 176-178 |
| **Discount rate** | 10 | Report the discount rate(s) and reason chosen. | NA |
| **Selection of outcomes** | 11 | Describe what outcomes were used as the measure(s) of benefit(s) and harm(s). | Page 6, Row 189 |
| **Measurement of outcomes** | 12 | Describe how outcomes used to capture benefit(s) and harm(s) were measured. | Page 5, Rows 126-155 |
| **Valuation of outcomes** | 13 | Describe the population and methods used to measure and value outcomes. | Pages 5- 6, Rows 156-171 |
| **Measurement and valuation of resources and costs** | 14 | Describe how costs were valued. | Page 6, Rows 172- 175 |
| **Currency, price date, and conversion** | 15 | Report the dates of the estimated resource quantities and unit costs, plus the currency and year of conversion. | Page 6, Rows 173-174 |
| **Rationale and description of model** | 16 | If modelling is used, describe in detail and why used. Report if the model is publicly available and where it can be accessed. | NA |
| **Analytics and assumptions** | 17 | Describe any methods for analysing or statistically transforming data, any extrapolation methods, and approaches for validating any model used. | Page 7, Rows 192-206 |
| **Characterising heterogeneity** | 18 | Describe any methods used for estimating how the results of the study vary for subgroups. | Page 7, Rows 201-206 |
| **Characterising distributional effects** | 19 | Describe how impacts are distributed across different individuals or adjustments made to reflect priority populations. | NA |
| **Characterising uncertainty** | 20 | Describe methods to characterise any sources of uncertainty in the analysis. | Page 7, Rows 194-195 |
| **Approach to engagement with patients and others affected by the study** | 21 | Describe any approaches to engage patients or service recipients, the general public, communities, or stakeholders (such as clinicians or payers) in the design of the study. | NA |
| **Results** |  |  |  |
| **Study parameters** | 22 | Report all analytic inputs (such as values, ranges, references) including uncertainty or distributional assumptions. | Page 7, Rows 217-223 |
| **Summary of main results** | 23 | Report the mean values for the main categories of costs and outcomes of interest and summarise them in the most appropriate overall measure. | Page 8, Rows 226-232 |
| **Effect of uncertainty** | 24 | Describe how uncertainty about analytic judgments, inputs, or projections affect findings. Report the effect of choice of discount rate and time horizon, if applicable. | NA |
| **Effect of engagement with patients and others affected by the study** | 25 | Report on any difference patient/service recipient, general public, community, or stakeholder involvement made to the approach or findings of the study | NA |
| **Discussion** |  |  |  |
| **Study findings, limitations, generalisability, and current knowledge** | 26 | Report key findings, limitations, ethical or equity considerations not captured, and how these could affect patients, policy, or practice. | Pages 8-10, Rows 245-280 |
| **Other relevant information** |  |  |  |
| **Source of funding** | 27 | Describe how the study was funded and any role of the funder in the identification, design, conduct, and reporting of the analysis | Page 1, Rows 18-22 |
| **Conflicts of interest** | 28 | Report authors conflicts of interest according to journal or International Committee of Medical Journal Editors requirements. | Page 1, Rows 24-26 |

*From:* Husereau D, Drummond M, Augustovski F, et al. Consolidated Health Economic Evaluation Reporting Standards 2022 (CHEERS 2022) Explanation and Elaboration: A Report of the ISPOR CHEERS II Good Practices Task Force. Value Health 2022;25. <doi:10.1016/j.jval.2021.10.008>
